# How can a change in the operating system of the mental health review board promote the discharge of long-term hospitalized psychiatric patients? A case study of Seoul city

**DOI:** 10.1186/1752-4458-8-33

**Published:** 2014-08-04

**Authors:** Myung-Soo Lee, Hee-Young Lim, Youngki Kim, Yong-Suk Lee

**Affiliations:** 1 Seoul Mental Health Center, Seoul, Republic of Korea; 2 Yongin Mental Hospital, Yongin, Republic of Korea; 3 Suji-Yonsei Psychiatric Clinic, Yongin, Republic of Korea; 4 Seoul Mental Health Center/Yongin Mental Hospital, 6 Bongeunsa-ro 21gil, Gangnam-gu, Seoul 135-545, Republic of Korea

**Keywords:** Length of hospital stay, Mental Health Review Board, Discharge order rate

## Abstract

**Background:**

One of the most typical and chronic problem in Korean mental health system is the prolonged length of hospital stay. In contrast to there are many components which leads to long length of stay of psychiatric patients in Korean situation such as low and fixed medical fee for psychiatric inpatient treatment, shortage of community resources, lack of care-givers’ awareness and so on, there are just few mechanisms to handle this issue such as Mental Health Review Board (MHRB) which is based on Mental Health Act since 1995. However, the discharge order rate was very low and there community care system after discharge order is still very weak.

**Case description:**

The Korean government has revised the Mental Health Act in 2008 and changed the operating principals of the MHRB from a regional level to a local level to strengthen the function of MHRB. However, the discharge order rate versus the whole evaluation requests still remains at a very low level or less than 5%. And it is still very difficult to execute a discharge order against a patient whose symptoms and conditions become psychiatrically stabilized enough for discharge, due to a shortage of community care facilities and a lack of social support system. These results are exactly same with former studies.

**Discussion:**

Any policies to promote psychiatric discharge including MHRB are needed to take the comprehensive factors into consideration, such as payment program, community infrastructure, increasing care-givers’ acceptance and so on.

**Conclusion:**

Despite of the political trial of Korean government to reduce length of stay of chronic psychiatric patients, it was not successful. Still it had failed to propose a detailed policy measure in terms of the above-mentioned prerequisites. Therefore, new system and program developments including reform of payment system which reflect prior studies’ recommendations are essential.

## Background

The most typical problem of the mental health system in Korea is the increasing trend in the number of psychiatric beds along with the prolonged length of hospital stay. The ratio of psychiatric beds per 1,000 persons stands at about 0.9, which is lower than that of Japan (2.8), Belgium (1.8) and Germany (1.3) [1]. However, considering the upward trend in psychiatric beds during the past decade, Korea is one of the few OECD member countries that have witnessed an increase in psychiatric beds. The average hospitalization stay is estimated to be approximately 150 days, which is 5 times longer than the average of the OECD member countries; moreover, if we observe the duration of hospitalization stay, 36.5% of patients experience long-term hospitalization for over 6 months [2]. Things are not much different in Seoul, where about one fifth of South Korea’s total population (50 million) reside. The average hospitalization stay of psychiatric patients in mental hospitals located in Seoul is estimated at 117 days, which is shorter than the national average, but still longer than the OECD average [2].

According to a study conducted in 1999 regarding the appropriateness of hospitalization [3], the rate of inappropriate hospital admissions was recorded at approximately 50%, which indicated that patients are hospitalized not only for medical reasons, but also for social reasons, including the absence of a caregiver or the absence of a residence. A national level study conducted ten years later in 2008 [4] revealed that the rate of inappropriate hospital admissions dropped to approximately 30%. Another study carried out by Seoul City in 2009 [5] displayed that the rate of hospital admissions for social reasons remained at about 30%, which was slightly improved compared to 10 years ago.

The aims of the Mental Health Act enacted in 1995 were to improve the treatment and management system of people with mental illness, to promote their human rights and to establish a community-based mental health system. As specified in the Mental Health Act, the operating system of the Mental Health Review Board (*MHRB*) conducts an evaluation every 6 months on the criteria such as continued hospitalization, discharge and treatment improvement, in order to decide whether a psychiatric patient should continue to be hospitalized or be discharged [6].

Until 2008, the MHRB had been operated on a regional level. However, the operation of only 16 regional MHRBs for the entire population of 50 million was inevitably faced with several realistic problems. The rate of discharge orders among those applicants who applied for continued hospitalization at the regional MHRBs across the country made up only 5% of the total requests for evaluation. Seoul has witnessed an improvement in the discharge order rate from 2.3% in 2004 to 6.7% in 2007, which was higher than the national average, but a rate that was still low [2].

Such a low discharge order rate can be ascribed to the following factors. Due to an excessive number of requests, continued hospitalization is decided through a paper evaluation rather than a face-to-face evaluation. Moreover, sufficient community resources are not available for those psychiatric patients who are clinically stabilized to be discharged, but who are without the support of caregivers. In addition, the after-care system for discharged patients is very weak. Further, although the review process is heavily dependent on a paper evaluation, the assessment of requests for continued hospitalization lacks objectivity. Finally, the legal responsibilities for accidents or incidents that may be engendered by a discharge order are not clearly defined [7].

In order to overcome such limitations of the system, the Korean government has revised the Mental Health Act in 2008 and changed the operating principals of the MHRB from a regional level to a local level. The revised act was to strengthen the localities of the MHRB, to improve access to psychiatric hospitals, to promote a face-to-face evaluation process, and to implement an after-care service in a more active manner. As a result, this change reflects the policy objectives to promote the return to a society of psychiatric patients who are hospitalized long-term for social reasons by boosting the current low discharge order rate.

This study aimed to find out the differences that the system and policy changes have made during the past 5 years after the revision of the Mental Health Act in terms of the operating outcomes of the Mental Health Review Board, of which the aim is to promote the discharge of psychiatric patients who are hospitalized for a long-term period.

### Description of case study

#### 1) Change in the operating system of the Mental Health Review Board in Seoul

**Figure 1:**
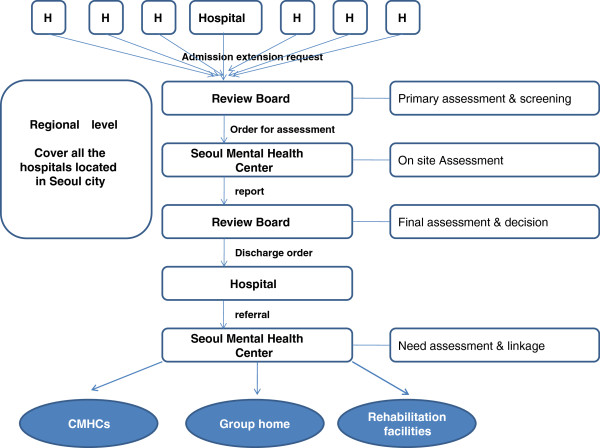
The operating system of Mental Health Review Board in Seoul city (~2008).

**Figure 2:**
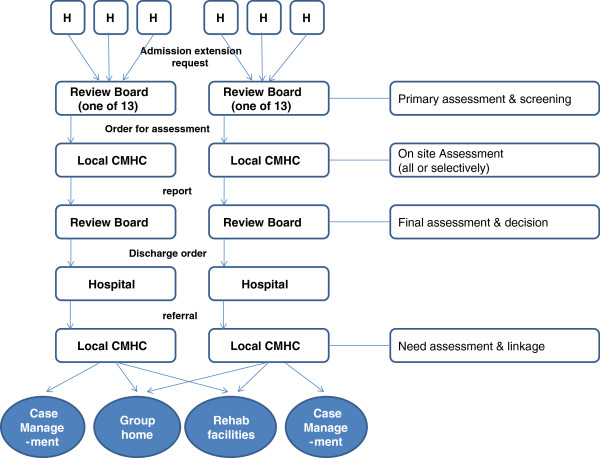
The revised operating system of Mental Health Review Board in Seoul city (2009~).

The operating system of the Mental Health Review Board of Seoul City prior to 2008 was as shown in Figure 1. The MHRB, which was operated on a regional level, received and evaluated requests for continued hospitalization of more than 6 months in order to narrow the list of candidates for discharge through the first round of paper evaluations. For the selected candidates, the mental health professionals of Seoul Mental Health Center (regional level) visited them in order to conduct a face-to-face interview evaluation; the professional then reported the results to the regional MHRB. The final decision was conducted based on the results of the interviews.Starting from 2009, the MHRB underwent a systemic change due to the revision of the Mental Health Act: one regional level MHRB in Seoul was divided and transferred to local level MHRBs in charge of 25 autonomous districts. Among the 25 districts in Seoul, 13 districts, where the psychiatric institutions are located, operated MHRB; the revised operating system is as shown in Figure 2.

Among the 13 local-level MHRBs, 11 districts have been found to conduct a face-to-face evaluation. 6 local MHRBs carried out a face-to-face evaluation for all candidates, whereas the remaining 5 performed a selective interview evaluation. The current face-to-face evaluation rate among the applicants for continued hospitalization well exceeds the average rate of the regional level MHRB (40%), which was operated until 2008.

#### 2) Number of requests for continued hospitalization and trend of discharge order rate in Seoul: 2008 ~ 2012

If we compare the past trends after and before the revision in the number of requests for continued hospitalization, the number of requests grew from 810 cases in 2008 to 1,034 cases in 2012. At that time, the number of psychiatric beds in Seoul was increased from 7,593 beds (0.74 bed per 1,000 people) to 8,154 beds (0.8 bed per 1,000 people). Therefore, it can be presumed that a hike in the requests for continued hospitalization was more influenced by an increase in psychiatric beds rather than the activity of the Mental Health Review Board. In contrast to this, the discharge order rate revealed a downward trend from 3.3% in 2009 to 2.5% in 2010; however, after a short-lived rebound to 5.2% in 2011, it fell again to 3.6%, which ultimately shows a decrease from 6.7% in 2008 (Table 1).

**Table 1:** The trend of psychiatric beds, evaluation cases of MHRB, and discharge order rate

	**2008**	**2009**	**2010**	**2011**	**2012**
Number of Psychiatric Beds (N)	7,593	7,738	7,773	7,659	8,154
Number of Evaluation Cases (N)	810	945	1,155	1,099	1,034
Rate of Discharge Order (%)	6.7	3.3	2.5	5.2	3.6

#### 3) Influential factors for discharge order

Seoul City has a total of 167 Mental Health Review Board members in 25 districts. In order to identify the major influential factors in the process of the discharge order, the study targeted and carried out a survey on 63 MHRB members from 13 districts, who were in charge of the paper and face-to-face evaluations, in order to decide on the requests for continued hospitalization or for legal matters, including discharge orders.

Those who were surveyed were comprised of 16 psychiatrists (25.4%), 13 mental health social workers (20.6%), 11 mental health nurses (17.5%), 9 lawyers (14.3%), 11 government officers (17.5%) and 2 caregivers of psychiatric patients (3.2%). The average tenure of these MHRB members was estimated at 26.9 months (S.D. = 17.4).

The most influential factor for the members was if the patient has the potential risk of self-injury or causing injury to others. The second most significant factor is the existence of a caregiver or a residence after discharge. In contrast, the length of hospital stay and the opinions of psychiatric patients or their caregivers are relatively not considered in the decision-making process (Table 2).

**Table 2:** Influencing factors to board members considering discharge order (5 point scale)

	**Mean**	**S.D.**	**Mode**
Risk of Self-injury or Injury to Others	4.90	.296	5
Eccentric Behaviors or Regression	3.86	.800	3
Unrealistic and Illogical Thinking	3.76	.893	3
Memory, Orientation and Judgment Impairment	3.56	.894	3
Risk of Discontinuation of Treatment after Discharge	3.94	1.030	4
With or Without Caregivers and Residences	4.50	.671	5
Access to Residential Facility	3.85	1.006	4
Available Facility in Patient’s Residence Area	3.61	1.077	4
Patient’s Opinion	3.21	0.936	3
Caregiver’s Opinion	3.17	1.027	3
Duration of Hospitalization Stay	3.06	1.190	4

Among the surveyed members of the MHRB, 92.9% respondents stated that the discharge order rate was low. 76.5% of the respondents mentioned their hardship to execute a discharge order. Among those difficulties, 93.3% of the respondents said that it was difficult to execute a discharge order in the case of a patient who was psychiatrically stable enough to be discharged, but was without a social support system. 60% of them cited that it was difficult to give a discharge order to a patient who was highly likely to be hospitalized again right after the discharge. Finally, 37.8% indicated that it was hard to execute a discharge order when a patient’s family members complained about it or refused to accept the order.

#### 4) Effectiveness of the revised Mental Health Review Board operating system

To make it simple, the problems revealed by the previous MHRB-related studies, before the transformation of the MHRB operating system, depicted almost similar patterns after the change. That is to say, the discharge order rate versus the whole evaluation requests still remains at a very low level or less than 5%. Moreover, the rate has become lower than prior to the change. And also it is still very difficult to execute a discharge order against a patient whose symptoms and conditions become psychiatrically stabilized enough for discharge, due to a shortage of community care facilities and a lack of social support system. These results are exactly same with former study [8]. MHRB still worries about high probability of readmission after discharge order like former study results; the rate of re-admission within one day after discharge hovers around 28%, while the ratio of re-admission within 7 days after discharge accounts for 33% of the entire discharged patients [9].

In conclusion, it is hard to say that the change in the operating system of the MHRB after the revision of the Mental Health Act in 2008 has served as a mechanism to improve the current status of long-term hospitalization; furthermore, it is not much to say that the current system is not successful.

## Discussion

In Korea, psychiatric admissions are categorized by four types. First one is voluntary admission which is done by patients’ request followed by agreement of physician. Second one is admission by two care-givers’ agreement with psychiatrists’ clinical decision, which is one of involuntary admission and all the cases of MHRB are from this type of admission. Third one is admission by director of local government, which also is involuntary admission focused on risks of self harm or to others. This type of admission has two consecutive components, which is admission for evaluation up to 14 days and followed by admission for treatment by the decision of two physician certificate up to maximum 3 months. The last type of admission is emergency admission done by agreement of police and physician up to 72 hours. In Korea there is no intervention mechanism of court in any type of admissions yet.

In a research report on the improvement of human rights of the mentally disabled [10], the National Human Rights Commission of Korea pointed out ‘long-term forced confinement’ as a serious breach of human rights. The use of the term ‘confinement’ instead of ‘admission’ for treatment can be interpreted as a distortion due to the complete misunderstanding of mental illnesses. Despite such factor, it is obviously true that psychiatric patients are admitted to a hospital not only for clinical reasons, but also for social reasons.

There are various factors that aggravate the long-term hospitalization of psychiatric patients in Korea [11-13]. What a more grave problem is the caregivers’ factor. Among the patients who are evaluated by psychiatrists as being sufficiently stabilized to be discharged, 13% of the cases were failed due to their caregivers’ refusal [14]. In addition, among those caregivers who repeatedly re-admitted the psychiatric patients to mental hospitals, 50% said that they would go to other hospitals in order to re-hospitalize them if the MHRB gave them a discharge order [15]. It can be said that this action was due to a lack of information about the provision of community services; however, it can also be interpreted as an attempt to avoid their responsibilities and burdens to care for the psychiatric patients.

Medicaid patients who pay a small copayment tend to be hospitalized for a long-term period. In the case of Medicaid patients, their patient families’ decisions are also strongly influenced by the following self-contradictory truth, that the economic burden of care-givers is higher when of community care than inpatient treatment. As such, South Korea does not have diverse mechanisms to change the current status of extended hospitalization. Thus, it can be said that without a payment policy based on the economic logic, the activity of the MHRB, in accordance with the Mental Health Act, is the only possible control mechanism.

This study was conducted with an aim to assess whether the revision of the Mental Health Act in 2008 had a profound impact on resolving the problems of the MHRB operating system, but which ended up with reconfirming the previous problems. Therefore, this study believed that Korea’s MHRB and other discharge promotion policies equivalent to it need to take the following factors into consideration. First of all, according to the study results, even though the clinical opinions of psychiatrists have a great impact on the evaluation of continued hospitalization of psychiatric patients, there is no compulsory enforcement nor any detailed document format that can compel psychiatrists to specify the clinical findings regarding the patients in detail. Although a face-to-face evaluation has been strengthened by the transformation of the regional MHRB into local MHRBs, a systemic tool needs to be established in order to encourage them to provide more detailed opinions, in that an objective and professional opinion of a psychiatrist who have treated a patient for more than 6 months can be a critical foundation for the decision-making. Second, although the members of the MHRB place great stress on the psychiatric conditions and symptoms in their evaluation process, the number one reason as to why they are unwilling to give a discharge order is the lack of a social support system for the after-care of psychiatric patients. After all, even though a patient is sufficiently stabilized to be discharged, it can become difficult to execute a discharge order if a social support system is not available. The World Health Organization presented a guideline, which stipulates that involuntary admission should be allowed only when the two prerequisites, such as the ‘Risk of self-injury or injury to others’ and ‘Clinical psychiatric evidence for need of in-patient care’ , are met [16]. That is to say, a patient should be discharged if the two conditions for admission are not met; however, the reality in Korea is far from it. It all boils down to the fact that the reinforcement of the community infrastructure to encourage the return of psychiatric patients to the society is the precondition for resolving long-term hospitalization.

City of Seoul set up the ‘Seoul Mental Health 2020 Plan’ in 2004 [17] and presented its goal of providing the badly-needed community infrastructure. 46% of the estimated service demand for residential facility was secured by Seoul City as of 2013. Yet, this study failed to assess how actively an attempt was made to affiliate psychiatric patients who were diagnosed to be stable enough for discharge with the residential service system. Simultaneously, this study also could not carry out an analysis on the existence of any barriers that blocked discharged psychiatric patients from entering the residential service system. From a residential service perspective, there is a need to re-define the priorities of patients for preferred residential service beneficiaries. At the same time, there is a need to reach an agreement on a policy attempt to concentrate on the case management service of the Community Mental Health Center (CMHC) on candidates for discharge by affiliating them with the residential service. Third, after the MHRB was transformed from a regional level into a local level, one of the newly emerging problems is that there is no clear clarification of who is responsible for managing and tracking the patients after discharge.

The mental hospitals in Korea are still new to the concept of ‘Catchment Area’. More than 85% of psychiatric services are provided by the private sector and a patient can be admitted to any psychiatric institutions outside of his or her own residence area. For example, given that half of the psychiatric patients whose legal residences are in Seoul City are currently hospitalized at psychiatric hospitals in other areas. In other words, in the case of psychiatric patients who are to be admitted to a hospital outside of their residence, there is no clear division of roles in the discharge process in terms of who will take responsibility for them. It appears that one of the possible solutions to resolve this problem is to strengthen the legal binding role of the CMHC as a public service provider. A report issued by the National Human Rights Commission of Korea [10] has suggested several measures, which includes granting a right to intervene at the time of admission and discharge and enacting a legislation to introduce the mandatory reporting of on-site evaluation in the case of involuntary admission.

The Korean government has included a proposal to shorten the first required period for evaluation for discharge from 6 months to 3 months in the 2014 amendment of the Mental Health Act. This acknowledges that there have been limitations in simply changing the operational mode of the MHRB, and at the same time, it can be considered as an alternative measure to promote early intensive care and early discharge.

## Conclusion

Despite of the political trial of Korean government to reduce length of stay of chronic psychiatric patients, it was not successful. Still it had failed to propose a detailed policy measure in terms of the above-mentioned prerequisites. Therefore, new system and program developments including reform of payment system which reflect prior studies’ recommendations are essential.

## Competing interests

The authors declare that they have no competing interests.

## Authors’ contributions

All authors participated in the design of the study and conceptualization of case report. MSLee made first draft and other authors made substantial edits. All authors read and approved the final manuscript.

## Authors’ information

M.S. Lee is a psychiatrist and has been playing a role in the community psychiatric setting as a director of mental health center and a board member of Mental Health Review board since last 10 years.

H.Y. Lim is a psychiatric social worker and has been working as a team manager in Seoul Mental Health Center. She has played role as a team leader of community research team.

Y. Kim is a 15 years career psychiatrist and working as a consultant to community mental health services.

Y.S. Lee is a psychiatrist working at mental hospital and he had played a role of director of community mental health center for about 3 years from 2009 to 2011.
